# Supplementary figures and images for: Agrochemical control of gene expression using evolved split RNA polymerase
Source: PeerJ. 2022 Jun 16;10:e13619. doi: 10.7717/peerj.13619 (PMC9206840; doi:10.7717/peerj.13619)

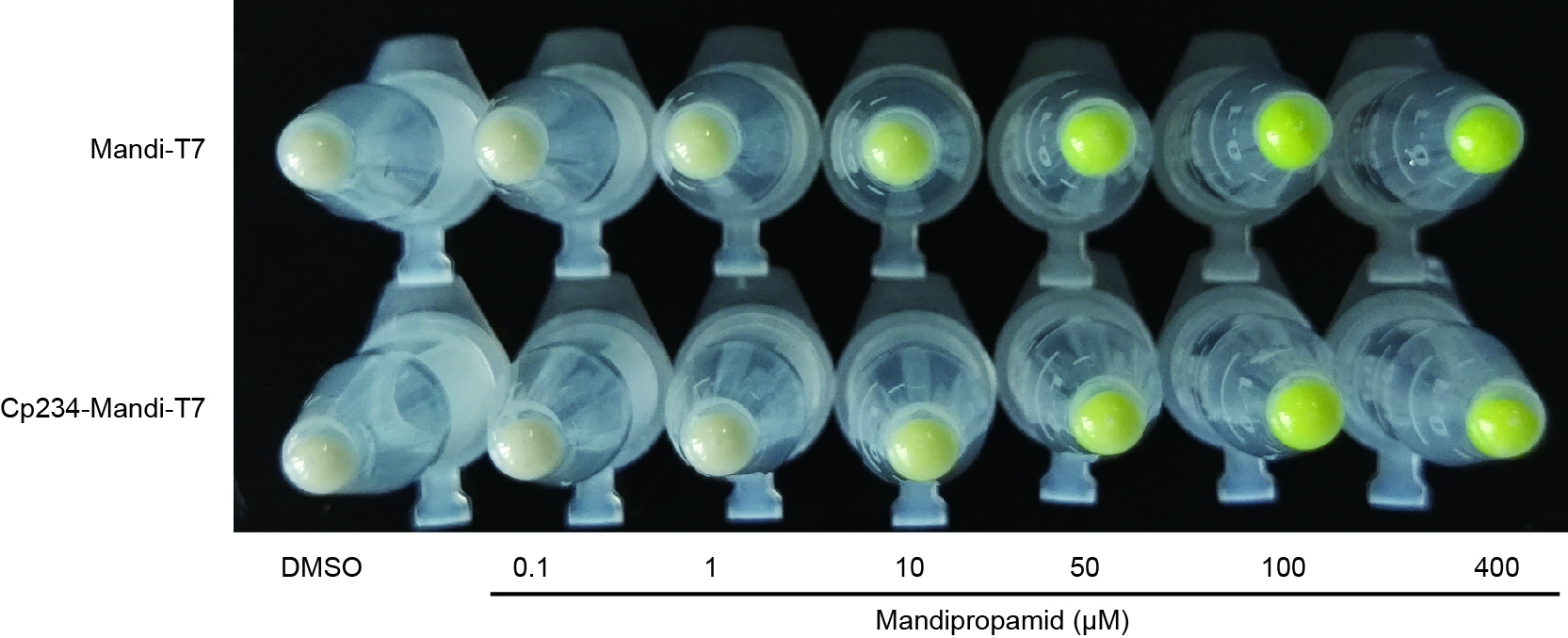

Supplement: Supplemental Information 2 — Picture was taken under normal day light condition. [file peerj-10-13619-s002.jpg]

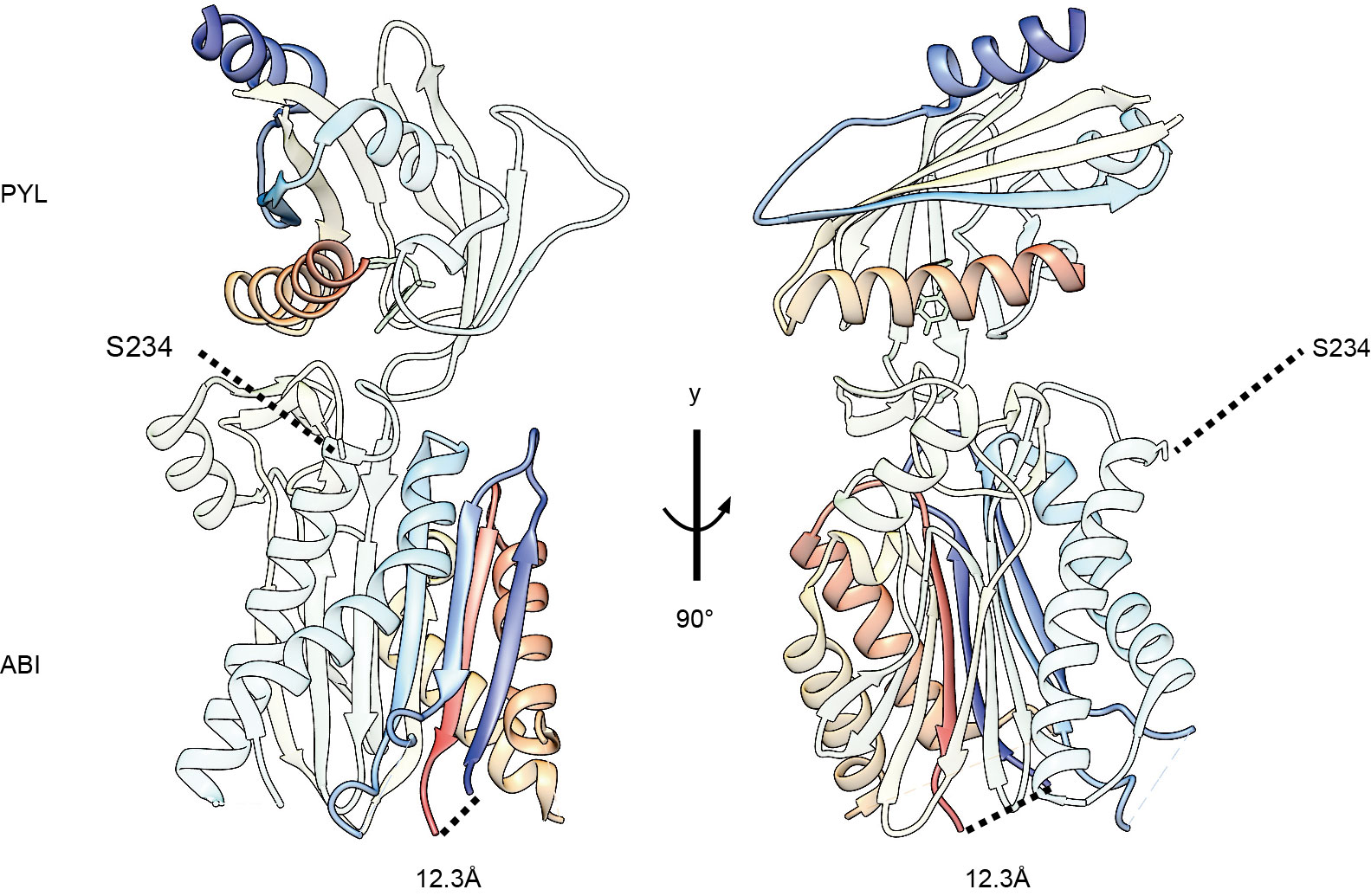

Supplement: Supplemental Information 5 — Peptide chains are shown in rainbow color from blue at the N terminus to red at the C terminus. The distance between original termini of ABI is 12.3 Å. The cartoon was generated based on the structure of ABI-PYL-Pyrabactin complex (PDB ID: 3nmn) using Chimera 1.15 [file peerj-10-13619-s005.jpg]

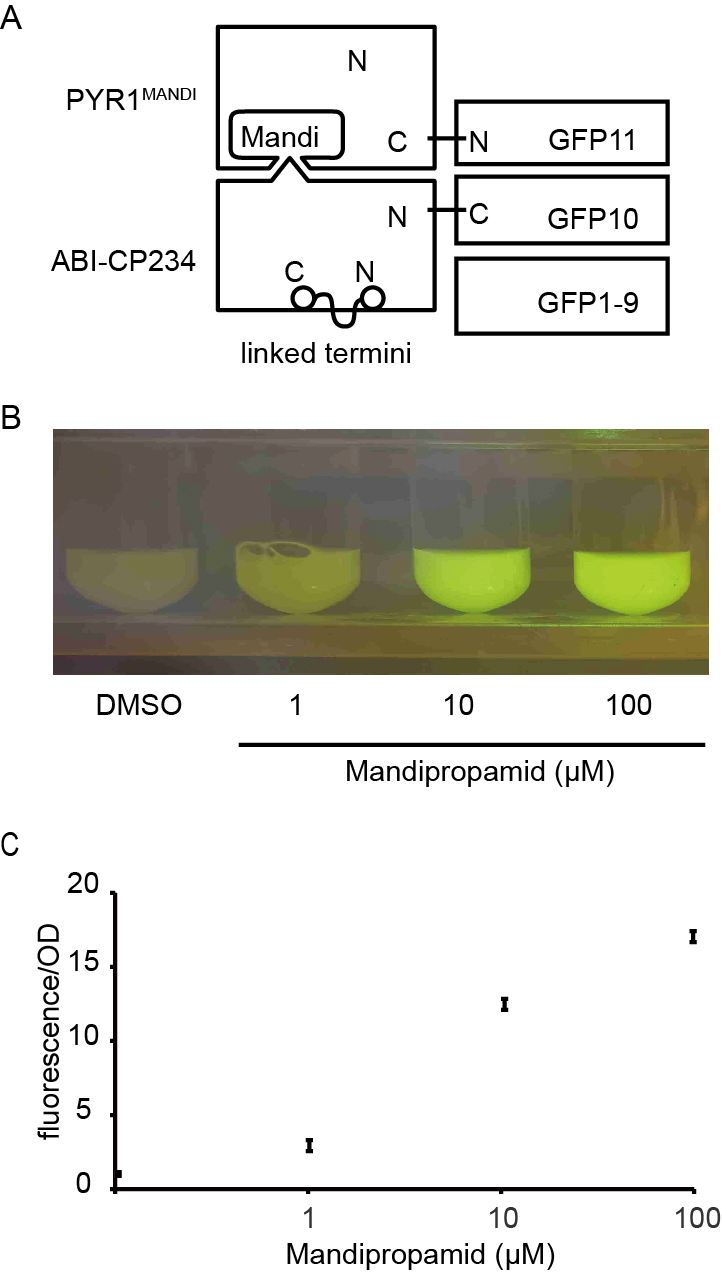

Supplement: Supplemental Information 6 — A, Schematic of the tripartite split-GFP assay. The original termini of ABI were linked by a flexible linker. GFP protein is reconstituted only if GFP10 and GFP 11 fragments are brought into close proximity. B, photo of E. coli liquid culture taken with orange filter illuminated by blue light. C, dose-response analysis of the tripartite split-GFP assay [file peerj-10-13619-s006.jpg]

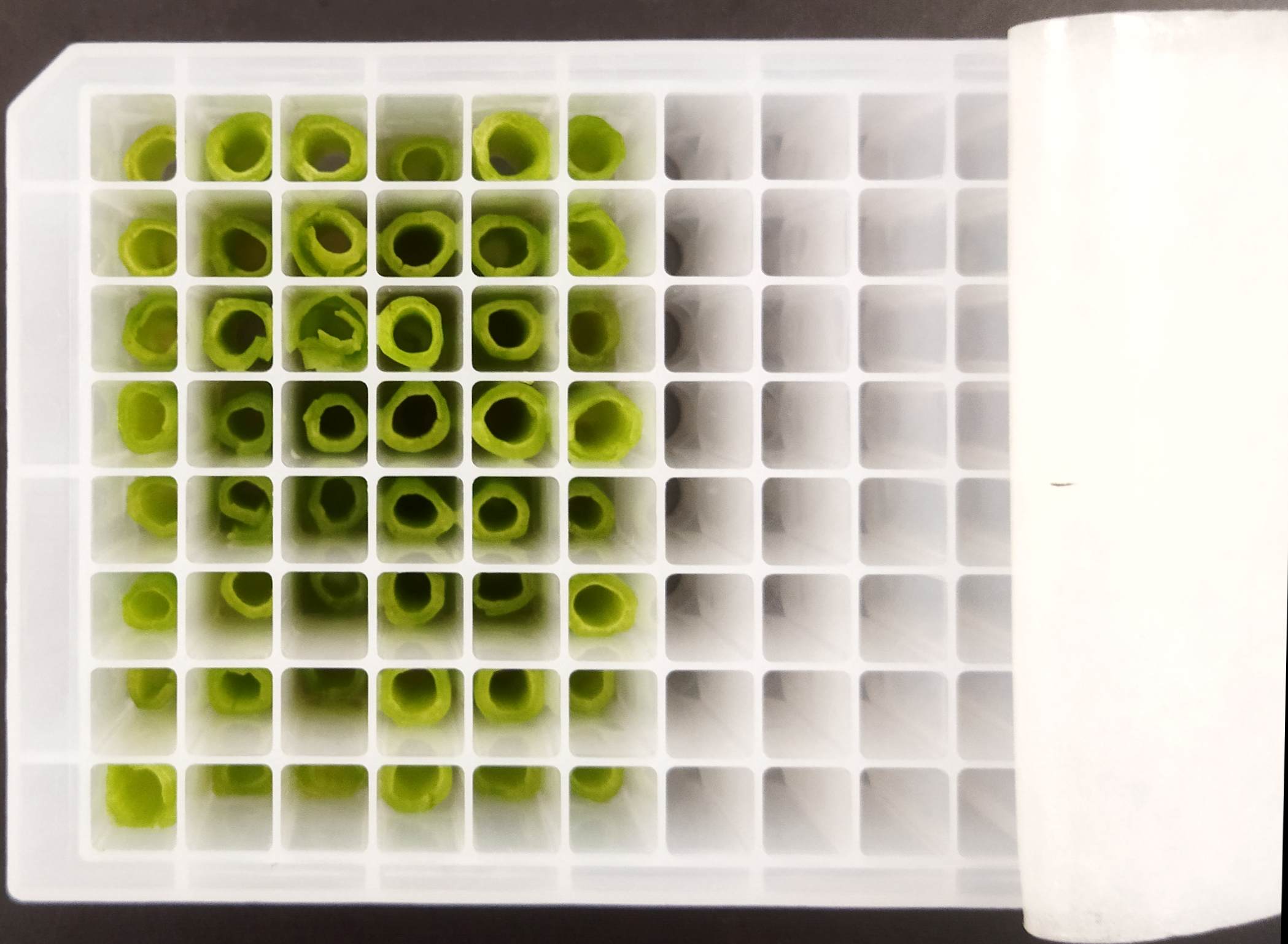

Supplement: Supplemental Information 7 [file peerj-10-13619-s007.jpg]

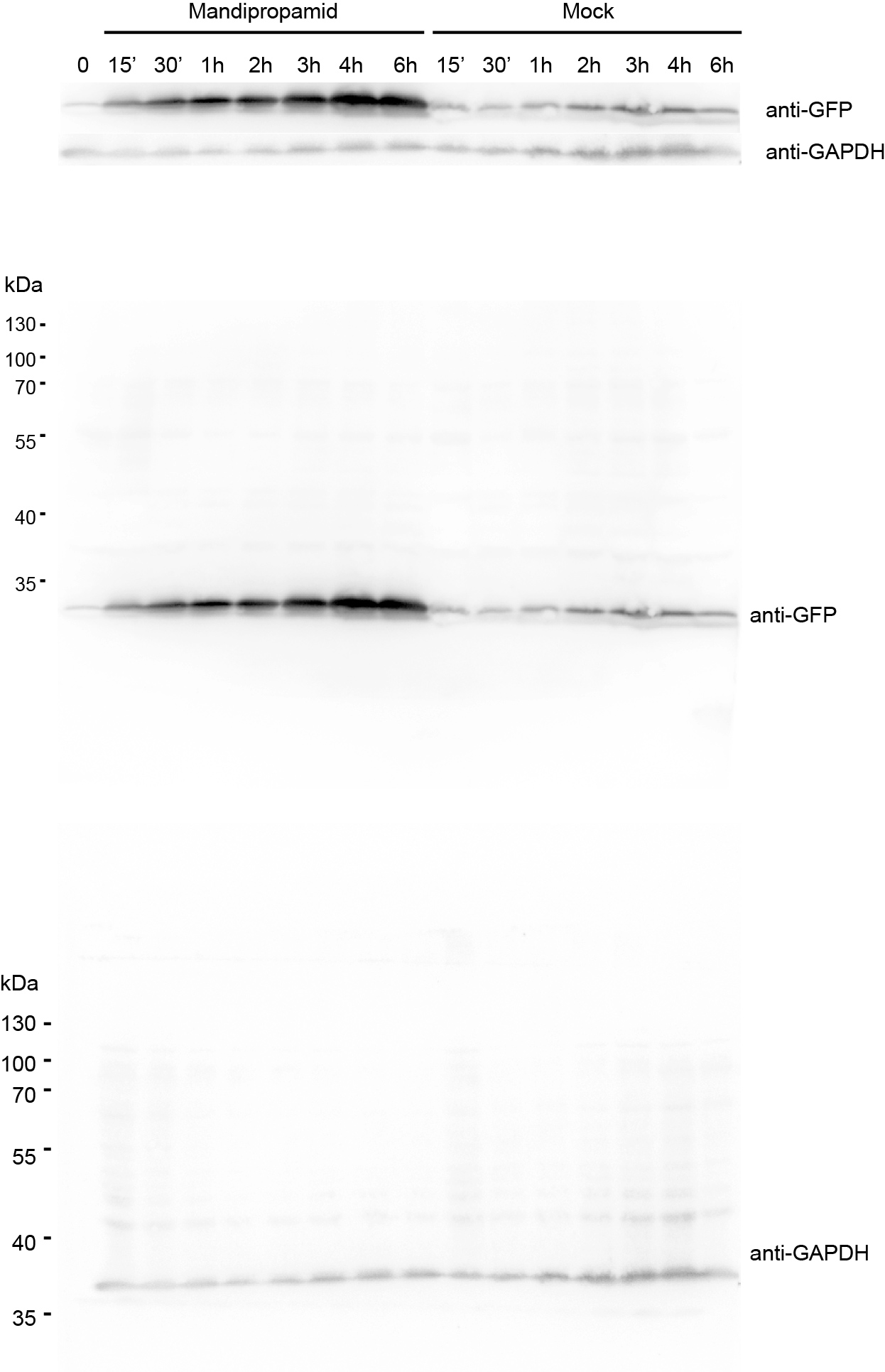

Supplement: Supplemental Information 8 [file peerj-10-13619-s008.jpg]

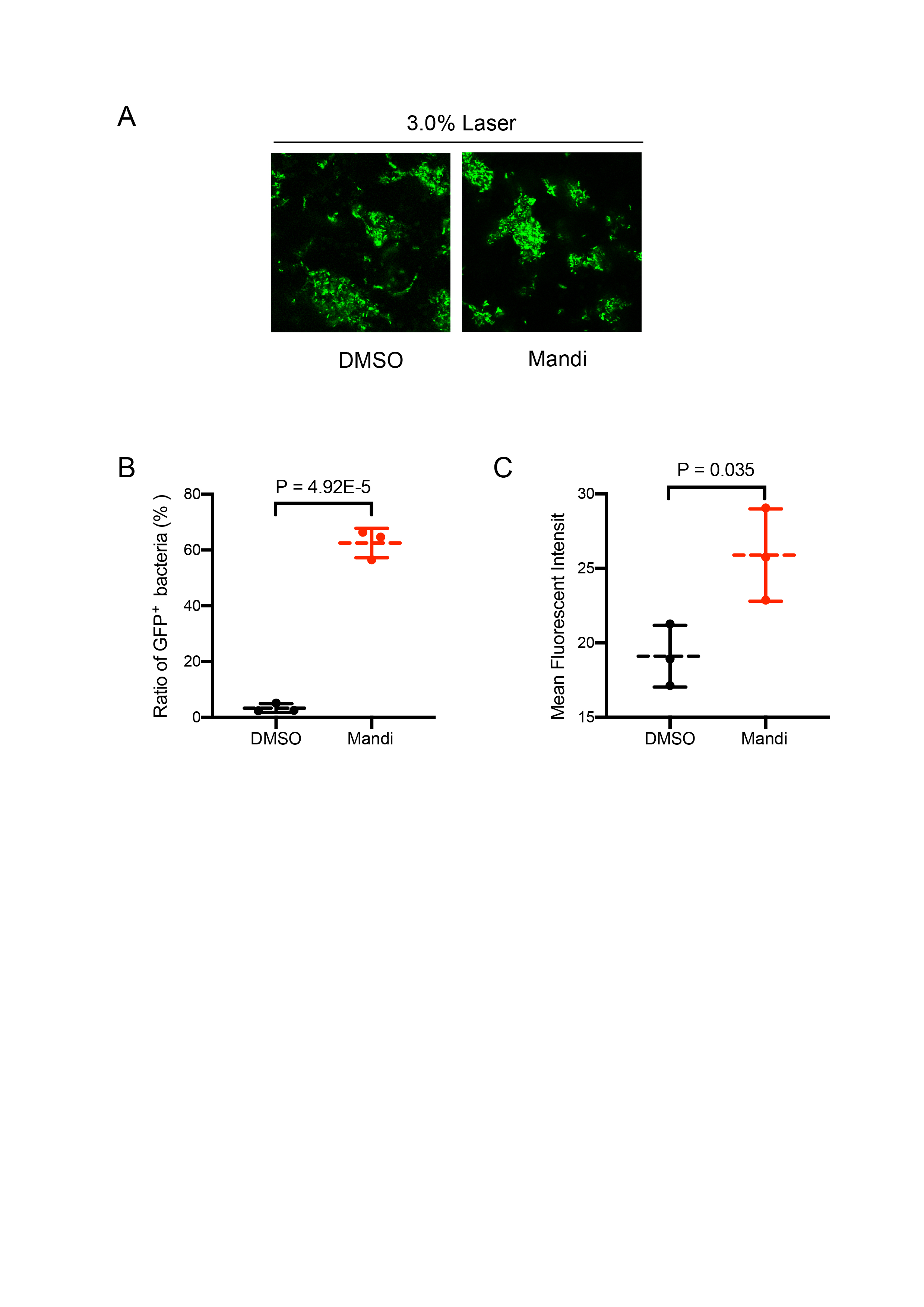

Supplement: Supplemental Information 9 — A, GFP fluorescence observed under high laser power (3%), the same region of interest as in Figure 1I. Bar: 50 µm. B, percentage of cells with GFP signal under low laser power (0.5%). Total cell number is determined under high laser power (3%). Results from three different positions were shown. C, Mean fluorescence intensity of GFP+ cells counted in B. [file peerj-10-13619-s009.jpg]

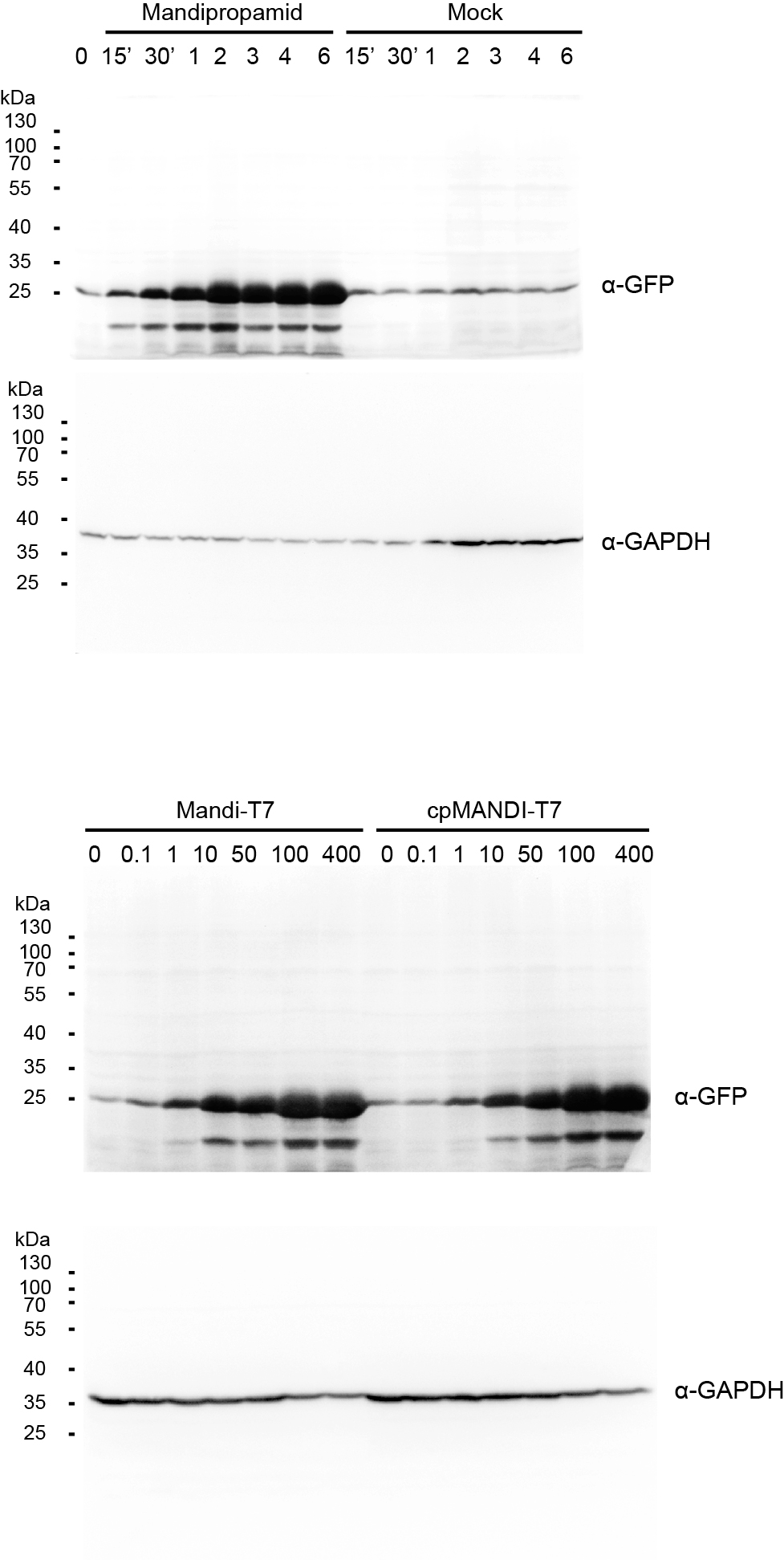

Supplement: Supplemental Information 10 [file peerj-10-13619-s010.jpg]
