# Supplementary material for: Agrochemical control of gene expression using evolved split RNA polymerase
Source: PeerJ. 2022 Jun 16;10:e13619. doi: 10.7717/peerj.13619 (PMC9206840; doi:10.7717/peerj.13619)
Supplement: Supplemental Information 11 [file peerj-10-13619-s011.docx]

Table S1: sequence and source of genetic parts

| Name | Sequence | Source |
| --- | --- | --- |
| P1 | tgttcacattcgaaccgtctctgctttgacatcttatgattctcgactgtaaagtcgtggcca | 1 |
| P2 | tgttcacattcgaaccgtctctgctttgacaacatgctgtgcggtgttgtaaagtcgtggccaggagaatacgacag | 1 |
| RBS1 | caacgctgcacccgaatcacattacggactattatt | 2 |
| RBS2 | gcaattgcaagaaggaggatattg | 2 |
| T7-eRNAPN (d5-19) | MNTINIAKNDFSDIELAAIPLNTLADHYGERSARGQLALEHESYEMGEARFRKMFECQLKAGKVADNAAAKPLITTLLPKMIARINDWFEEVKAKRGRRPTAFKFLKEIKPEAVAYITIKTSLACLTSADNTTVQAVASAIGRTIEDEARFGRIRDLEAKHFKKNVEEQLNKRVGHVYK | 3 |
| ABI | VPLYGFTSICGRRPEMEAAVSTIPRFLQSSSGSMLDGRFDPQSAAHFFGVYDGHGGSQVANYCRERMHLALAEEIAKEKPMLCDGDTWLEKWKKALFNSFLRVDSEIESVAPETVGSTSVVAVVFPSHIFVANCGDSRAVLCRGKTALPLSVDHKPDREDEAARIEAAGGKVIQWNGARVFGVLAMSRSIGDRYLKPSIIPDPEVTAVKRVKEDDCLILASDGVWDVMTDEEACEMARKRILLWHKKNAVAGDASLLADERRKEGKDPAAMSAAEYLSKLAIQRGSKDNISVVVVDLK (AT4G26080.1, V126-K423, D143A) | 3, 4 |
| ABI-CP234  (circularly permutated) | SVAPETVGSTSVVAVVFPSHIFVANCGDSRAVLCRGKTALPLSVDHKPDREDEAARIEAAGGKVIQWNGARVFGVLAMSRSIGDRYLKPSIIPDPEVTAVKRVKEDDCLILASDGVWDVMTDEEACEMARKRILLWHKKNAVAGDASLLADERRKEGKDPAAMSAAEYLSKLAIQRGSKDNISVVVVDLKGGSGSGSSVPLYGFTSICGRRPEMEAAVSTIPRFLQSSSGSMLDGRFDPQSAAHFFGVYDGHGGSQVANYCRERMHLALAEEIAKEKPMLCDGDTWLEKWKKALFNSFLRVDSEIE  (S234 -K423-linker-V126-E233, D143A) | 3 |
| Terminator 1 | attcaagacccccgcaccgaaaggtccgggggttttttttacta | 5 |
| PYR1^MANDI^ | MPSELTPEERSELKNSIAEFHTYQLDPGSCSSLHAQRIHAPPELVWSIVRRFDKPQTHRHFIKSCSVEQNFEMRVGCTRDIIVISGLPANTSTERLDILDDERRVTGASIIGGEHRLTNYKGVTTVHRFEKENRIWTVVLESYVVDMPEGNSEDDTRMLADTVVKLNLQKLATVAEAMA | 6 |
| T7-RNAPC | KAFMQVVEADMLSKGLLGGEAWSSWHKEDSIHVGVRCIEMLIESTGMVSLHRQNAGVVGQDSETIELAPEYAEAIATRAGALAGISPMFQPCVVPPKPWTGITGGGYWANGRRPLALVRTHSKKALMRYEDVYMPEVYKAINIAQNTAWKINKKVLAVANVITKWKHCPVEDIPAIEREELPMKPEDIDMNPEALTAWKRAAAAVYRKDKARKSRRISLEFMLEQANKFANHKAIWFPYNMDWRGRVYAVSMFNPQGNDMTKGLLTLAKGKPIGKEGYYWLKIHGANCAGVDKVPFPERIKFIEENHENIMACAKSPLENTWWAEQDSPFCFLAFCFEYAGVQHHGLSYNCSLPLAFDGSCSGIQHFSAMLRDEVGGRAVNLLPSETVQDIYGIVAKKVNEILQADAINGTDNEVVTVTDENTGEISEKVKLGTKALAGQWLAYGVTRSVTKRSVMTLAYGSKEFGFRQQVLEDTIQPAIDSGKGLMFTQPNQAAGYMAKLIWESVSVTVVAAVEAMNWLKSAAKLLAAEVKDKKTGEILRKRCAVHWVTPDGFPVWQEYKKPIQTRLNLMFLGQFRLQPTINTNKDSEIDAHKQESGIAPNFVHSQDGSHLRKTVVWAHEKYGIESFALIHDSFGTIPADAANLFKAVRETMVDTYESCDVLADFYDQFADQLHESQLDKMPALPAKGNLNLRDILESDFAFA* | 3 |
| Terminator 2 | ctagcataaccccttggggcctctaaacgggtcttgaggggttttttg | pET23a |
| GFP (sfGFP) | MRKGEELFTGVVPILVELDGDVNGHKFSVRGEGEGDATNGKLTLKFICTTGKLPVPWPTLVTTLTYGVQCFARYPDHMKQHDFFKSAMPEGYVQERTISFKDDGTYKTRAEVKFEGDTLVNRIELKGIDFKEDGNILGHKLEYNFNSHNVYITADKQKNGIKANFKIRHNVEDGSVQLADHYQQNTPIGDGPVLLPDNHYLSTQSVLSKDPNEKRDHMVLLEFVTAAGITHGMDELYK | 1 |
| T7 pro_RBS  (for sfGFP) | taatacgactcactatagggagaccacaacggtttccctctaCaaataattttgtttaactttaagaaggagatatacat | pET23a |
| GFP10 | MDLPDDHYLSTQTILSKDLN | 7 |
| GFP11 | EKRDHMVLLEYVTAAGITDAS | 7 |
| GFP1-9 | MRKGEELFTGIVPILVELDGDVNGHKFFVRGEGEGDATIGKLSLKFICTTGKLPVPWPTLVTTLTYGVQCFSRYPDHMKRHDFFKSAMPEGYVQERTIYFKDDGTYKTRAEVKFEGDTLVNRIELKGIDFKEDGNILGHKLEYNFNSHKVYITADKQNNGIKANFTIRHNVEDGSVQLADHYQQNTPIGDGPVLLP | 7 |
| T7_lacO_RBS  (for GFP1-9) | taatacgactcactataggggaattgtgagcggataacaattcccctctagaaataattttgtttaactttaagaaggagatatacc | pET28a |
| J23109 | tttacagctagctcagtcctagggactgtgctagct | Biobrick |
| J23105 | tttacggctagctcagtcctaggtactatgctagct | Biobrick |
| mcherry | MVSKGEEDNMAIIKEFMRFKVHMEGSVNGHEFEIEGEGEGRPYEGTQTAKLKVTKGGPLPFAWDILSPQFMYGSKAYVKHPADIPDYLKLSFPEGFKWERVMNFEDGGVVTVTQDSSLQDGEFIYKVKLRGTNFPSDGPVMQKKTMGWEASSERMYPEDGALKGEIKQRLKLKDGGHYDAEVKTTYKAKKPVQLPGAYNVNIKLDITSHNEDYTIVEQYERAEGRHSTGGMDELYK |  |

References

1, Bai C, Zhang Y, Zhao X, *et al.* Exploiting a precise design of universal synthetic modular regulatory elements to unlock the microbial natural products in Streptomyces. Proceedings of the National Academy of Sciences of the United States of America, 2015, 112(39):12181-12186. doi: 10.1073/pnas.1511027112.

2, Salis HM, Mirsky EA, Voigt CA. Automated design of synthetic ribosome binding sites to control protein expression. Nature Biotechnology, 2009, 27(10):946-950. doi: 10.1038/nbt.1568.

3, Pu J, Kentala K, Dickinson BC. Multidimensional Control of Cas9 by Evolved RNA Polymerase-Based Biosensors. Acs Chemical Biology, 2018, 13(2):431-437. doi: 10.1021/acschembio.7b00532.

4, Liang FS, Ho WQ, Crabtree GR. Engineering the ABA plant stress pathway for regulation of induced proximity. Sci Signal. 2011 Mar 15;4(164):rs2. doi: 10.1126/scisignal.2001449. PMID: 21406691; PMCID: PMC3110149.

5, Reis AC, Halper S M, Vezeau GE, *et al.* Simultaneous repression of multiple bacterial genes using nonrepetitive extra-long sgRNA arrays. Nature Biotechnology, 2019, 37(11):1294-1301.

6, Park, SY, Peterson, *et al.* Agrochemical control of plant water use using engineered abscisic acid receptors. Nature, 2015, 520(7548):545-548. doi: 10.1038/nature14123.

7, Pedelacq JD, Waldo GS, Cabantous S. High-Throughput Protein-Protein Interaction Assays Using Tripartite Split-GFP Complementation. Methods Molecular Biology. 2019, 2025:423-437. doi: 10.1007/978-1-4939-9624-7_20.
