# Supplementary material for: Agrochemical control of gene expression using evolved split RNA polymerase
Source: PeerJ. 2022 Jun 16;10:e13619. doi: 10.7717/peerj.13619 (PMC9206840; doi:10.7717/peerj.13619)
Supplement: Supplemental Information 12 [file peerj-10-13619-s012.docx]

Table S2: sequence of plasmids

| pCDF-T7-sfGFP  (Turquoise:  T7 promoter  yellow:  sfGFP  purple: Terminator) | gcgaaattaatacgactcactatagggagaccacaacggtttccctctaCaaataattttgtttaactttaagaaggagatatacatatgcgtaaaggcgaagagctgttcactggtgtcgtccctattctggtggaactggatggtgatgtcaacggtcataagttttccgtgcgtggcgagggtgaaggtgacgcaactaatggtaaactgacgctgaagttcatctgtactactggtaaactgccagtaccttggccgactctggtaacgacgctgacttatggtgttcagtgctttgctcgttatccggaccacatgaagcagcatgacttcttcaagtccgccatgccggaaggctatgtgcaggaacgcacgatttcatttaaggatgacggcacgtacaaaacgcgtgcggaagtgaaatttgaaggcgataccctggtaaaccgcattgagctgaaaggcattgactttaaagaagacggcaatatcctgggccataagctggaatacaattttaacagccacaatgtttacatcaccgccgataaacaaaaaaatggcattaaagcgaatttcaaaattcgccacaacgtggaggatggcagcgtgcagctggctgatcactaccagcaaaacactccaatcggtgatggtcctgttctgctgccagacaatcactatctgagcacgcaaagcgttctgtctaaagatccgaacgagaaacgcgatcacatggttctgctggagttcgtaaccgcagcgggcatcacgcacggtatggatgaactgtacaaatgagaattcttgttcagaacgctcggtcttgcacaccgggcgttttttctttgtgagtccaatccaaaacgccgcgttcagcggcgttttttctgcttctgaaag |
| --- | --- |
| pJM135A  (Turquoise:  P1  Yellow:  eT7N-ABI  red:  Terminator 1  Teal:  J23105  Green:  PYR1^MANDI^-T7C  Blue:  Terminator 2  ) | tgttcacattcgaaccgtctctgctttgacatcttatgattctcgactgtaaagtcgtggccacaacgctgcacccgaatcacattacggactattattatgaacacgattaacatcgctaagaacgacttctctgacatcgaactggctgctatcccgctcaacactctggctgaccattacggtgagcgttcagctcgcggacagttggcccttgagcatgagtcttacgagatgggtgaagcacgcttccgcaagatgtttgagtgtcaacttaaagctggtaaggttgcggataacgctgccgccaagcctctcatcactaccctcctccctaagatgattgcccgcatcaacgactggtttgaggaagtgaaagctaagcgcggcaggcgcccgacagccttcaagttcctcaaagaaatcaagccggaagccgtagcgtacatcaccattaagacctctctggcttgcctcaccagtgctgacaatacaaccgttcaggctgtagcaagcgcaatcggtcggaccattgaggacgaggctcgcttcggtcgtatccgtgaccttgaagctaagcacttcaagaaaaacgttgaggaacaactcaacaagcgcgtagggcacgtctacaagggtggatccggctctggttcgagtgtccccctgtatgggttcaccagcatttgtgggcggagaccagagatggaggcagccgtcagcactattccccggttcctgcagtcaagctccggcagcatgctggacgggaggttcgatccacagtccgccgctcacttctttggggtctacgatggacatggcgggtcccaggtggccaactattgcagggagcgcatgcacctggctctggcagaggaaatcgccaaggagaaacctatgctgtgcgacggagatacatggctggaaaagtggaagaaagccctgttcaactcttttctgcgggtggactccgagatcgaatctgtcgctcctgagaccgtgggctctacaagtgtggtcgcagtggtcttcccatctcacatttttgtcgcaaattgcggcgacagccgggccgtgctgtgcaggggaaagaccgccctgccactgagcgtggaccataaacccgacagggaggatgaagcagcccgcatcgaggctgcaggaggcaaagtgatccagtggaacggagcacgggtgtttggcgtcctggccatgtcacgcagcattggggatcgatacctgaaaccatcaatcattcccgaccctgaagtgactgccgtcaagagagtgaaagaagacgattgcctgatcctggctagcgacggcgtctgggatgtgatgaccgacgaggaagcatgtgagatggcccgaaagcggattctgctgtggcataagaaaaatgccgtggctggcgatgctagtctgctggcagacgagcggagaaaggaagggaaagatcccgccgctatgagtgcagccgaatatctgtcaaaactggctattcagaggggcagtaaggacaacatctccgtcgtcgtcgtggacctgaagtgaggtaccattcaagacccccgcaccgaaaggtccgggggttttttttactatttaaatcctgccagtttacggctagctcagtcctaggtactatgctagctgcaattgcaagaaggaggatattgatgccatcggaacttacaccggaggaaaggtccgagttgaagaacagcattgcggagtttcatacatatcaactcgatccggggagttgttctagtttgcacgcccaaaggatacacgccccaccggaacttgtgtggagtatcgtccgccggttcgataaaccacaaacacacaggcatttcattaaaagctgctccgtggagcagaactttgaaatgagagtgggatgcacacgggatataatagttataagcggcctcccggccaacacgagcaccgaaagattggacatactggatgacgagcgccgcgtgacgggggcatcaataatcggtggggaacatcgccttacgaattataaaggcgtcactactgttcatagatttgagaaggaaaatagaatctggactgttgttctggaatcttatgtggttgatatgccagagggtaactcggaagatgatacacgcatgctggcggacacggttgtgaaacttaatctgcaaaagctggcaactgtcgccgaagccatggcatcaggatctggtagcgcaggatcaggctcgagtggtaaagcatttatgcaagttgtcgaggctgacatgctctctaagggtctcctcggtggcgaggcgtggtcttcgtggcataaggaagactctattcatgtaggagtacgctgcatcgagatgctcattgagtcaaccggaatggttagcctccaccgccaaaatgctggcgtagtaggtcaagactctgagactatcgaactcgcacctgaatacgctgaggctatcgcaacccgtgcaggtgcgctggctggcatctctccgatgttccaaccttgcgtagttcctcctaagccgtggactggcattactggtggtggctattgggctaacggtcgtcgtcctctggcgctggtgcgtactcacagtaagaaagcactgatgcgctacgaagacgtttacatgcctgaggtgtacaaagcgattaacattgcgcaaaacaccgcatggaaaatcaacaagaaagtcctcgcggtcgccaacgtaatcaccaagtggaagcattgtccggtcgaggacatccctgcgattgagcgtgaagaactcccgatgaaaccggaagacatcgacatgaatcctgaggctctcaccgcgtggaaacgtgctgccgctgctgtgtaccgcaaggacaaggctcgcaagtctcgccgtatcagccttgagttcatgcttgagcaagccaataagtttgctaaccataaggccatctggttcccttacaacatggactggcgcggtcgtgtttacgctgtgtcaatgttcaacccgcaaggtaacgatatgaccaaaggactgcttacgctggcgaaaggtaaaccaatcggtaaggaaggttactactggctgaaaatccacggtgcaaactgtgcgggtgtcgataaggttccgttccctgagcgcatcaagttcattgaggaaaaccacgagaacatcatggcttgcgctaagtctccactggagaacacttggtgggctgagcaagattctccgttctgcttccttgcgttctgctttgagtacgctggggtacagcaccacggcctgagctataactgctcccttccgctggcgtttgacgggtcttgctctggcatccagcacttctccgcgatgctccgagatgaggtaggtggtcgcgcggttaacttgcttcctagtgaaaccgttcaggacatctacgggattgttgctaagaaagtcaacgagattctccaagcagacgcaatcaatgggaccgataacgaagtagttaccgtgaccgatgagaacactggtgaaatctctgagaaagtcaagctgggcactaaggcactggctggtcaatggctggcttacggtgttactcgcagtgtgactaagcgttcagtcatgacgctggcttacgggtccaaagagttcggcttccgtcaacaagtgctggaagataccattcagccagctattgattccggcaagggtctgatgttcactcagccgaatcaggctgctggatacatggctaagctgatttgggaatctgtgagcgtgacggtggtagctgcggttgaagcaatgaactggcttaagtctgctgctaagctgctggctgctgaggtcaaagataagaagactggagagattcttcgcaagcgttgcgctgtgcattgggtaactcctgatggtttccctgtgtggcaggaatacaagaagcctattcagacgcgcttgaacctgatgttcctcggtcagttccgcctccagcctaccattaacaccaacaaagatagcgagattgatgcacacaaacaggagtctggtatcgctcctaactttgtacacagccaagacggtagccaccttcgtaagactgtagtgtgggcacacgagaagtacggaatcgaatcttttgcactgattcacgactccttcggtaccattccggctgacgctgcgaacctgttcaaagcagtgcgcgaaactatggttgacacatatgagtcttgtgatgtactggctgatttctacgaccagttcgctgaccagttgcacgagtctcaattggacaaaatgccagcacttccggctaaaggtaacttgaacctccgtgacatcctcgagtcggacttcgcgttcgcgtaatagcaccaccaccaccaccactgagatccggctgctaacaaagcccgaaaggaagctgagttggctgctgccaccgctgagcaataactagcataaccccttggggcctctaaacgggtcttgaggggttttttg |
| pCDF-T7-lacO-GFP1-9  (Turquoise:  T7 promoter  Red:  lacO  yellow:  GFP1-9  Violet:  terminator) | TAATACGACTCACTATAGGGGAATTGTGAGCGGATAACAATTCCCCTCTAGAAATAATTTTGTTTAACTTTAAGAAGGAGATATACCatgcgcaaaggcgaagaactgtttaccggcattgtgccgattctggtggaactggatggcgatgtgaacggccataaattttttgtgcgcggcgaaggcgaaggcgatgcgaccattggcaaactgagcctgaaatttatttgcaccaccggcaaactgccggtgccgtggccgaccctggtgaccaccctgacctatggcgtgcagtgctttagccgctatccggatcatatgaaacgccatgatttttttaaaagcgcgatgccggaaggctatgtgcaggaacgcaccatttattttaaagatgatggcacctataaaacccgcgcggaagtgaaatttgaaggcgataccctggtgaaccgcattgaactgaaaggcattgattttaaagaagatggcaacattctgggccataaactggaatataactttaacagccataaagtgtatattaccgcggataaacagaacaacggcattaaagcgaactttaccattcgccataacgtggaagatggcagcgtgcagctggcggatcattatcagcagaacaccccgattggcgatggcccggttcttcttccttaggaattcttgttcagaacgctcggtcttgcacaccgggcgttttttctttgtgagtcca |
| pJM134  (Turquoise:  J23101  Yellow:  G10- ABI-CP1  Red:  Terminator 1  Violet:  P2  Green:  PYR1^MANDI^-G11  Gray:  Terminator 2) | tttacagctagctcagtcctaggtattatgctagcgaaattacccttgccttaactaataagagagctgtctatggacctgcctgacgaccactacctgtccacccagaccatcctgtccaaggacctgaacggatctggtggatccggctctggttcgagttctgtcgctcctgagaccgtgggctctacaagtgtggtcgcagtggtcttcccatctcacatttttgtcgcaaattgcggcgacagccgggccgtgctgtgcaggggaaagaccgccctgccactgagcgtggaccataaacccgacagggaggatgaagcagcccgcatcgaggctgcaggaggcaaagtgatccagtggaacggagcacgggtgtttggcgtcctggccatgtcacgcagcattggggatcgatacctgaaaccatcaatcattcccgaccctgaagtgactgccgtcaagagagtgaaagaagacgattgcctgatcctggctagcgacggcgtctgggatgtgatgaccgacgaggaagcatgtgagatggcccgaaagcggattctgctgtggcataagaaaaatgccgtggctggcgatgctagtctgctggcagacgagcggagaaaggaagggaaagatcccgccgctatgagtgcagccgaatatctgtcaaaactggctattcagaggggcagtaaggacaacatctccgtcgtcgtcgtggacctgaagggcggtagtggttcgggttcttcggtccccctgtatgggttcaccagcatttgtgggcggagaccagagatggaggcagccgtcagcactattccccggttcctgcagtcaagctccggcagcatgctggacgggaggttcgatccacagtccgccgctcacttctttggggtctacgatggacatggcgggtcccaggtggccaactattgcagggagcgcatgcacctggctctggcagaggaaatcgccaaggagaaacctatgctgtgcgacggagatacatggctggaaaagtggaagaaagccctgttcaactcttttctgcgggtggactccgagatcgaatgaggtaccattcaagacccccgcaccgaaaggtccgggggttttttttactatttaaatcctgccagtgttcacattcgaaccgtctctgctttgacaacatgctgtgcggtgttgtaaagtcgtggccaggagaatacgacaggcaattgcaagaaggaggatattgatgccatcggaacttacaccggaggaaaggtccgagttgaagaacagcattgcggagtttcatacatatcaactcgatccggggagttgttctagtttgcacgcccaaaggatacacgccccaccggaacttgtgtggagtatcgtccgccggttcgataaaccacaaacacacaggcatttcattaaaagctgctccgtggagcagaactttgaaatgagagtgggatgcacacgggatataatagttataagcggcctcccggccaacacgagcaccgaaagattggacatactggatgacgagcgccgcgtgacgggggcatcaataatcggtggggaacatcgccttacgaattataaaggcgtcactactgttcatagatttgagaaggaaaatagaatctggactgttgttctggaatcttatgtggttgatatgccagagggtaactcggaagatgatacacgcatgctggcggacacggttgtgaaacttaatctgcaaaagctggcaactgtcgccgaagccatggcaggatcaggctcgagtggttcaggatctggtgcaagcgaaaagcgagaccatatggttttgcttgagtatgttacagcggctggcattaccgatgcatcatagtcggacttcgcgttcgcgtaatagcaccaccaccaccaccactgagatccggctgctaacaaagcccgaaaggaagctgagttggctgctgccaccgctgagcaataactagcataaccccttggggcctctaaacgggtcttgaggggttttttg |
| pJM1B6  (turquoise:  P1  Yellow:  eT7N- ABI_CP234  Red:  Terminator 1  Teal:  P2  Green:  PYR1^MANDI^-T7C  Blue:  Terminator 2  ) | ctatactcggatatctgttcacattcgaaccgtctctgctttgacatcttatgattctcgactgtaaagtcgtggccacaacgctgcacccgaatcacattacggactattattatgaacacgattaacatcgctaagaacgacttctctgacatcgaactggctgctatcccgctcaacactctggctgaccattacggtgagcgttcagctcgcggacagttggcccttgagcatgagtcttacgagatgggtgaagcacgcttccgcaagatgtttgagtgtcaacttaaagctggtaaggttgcggataacgctgccgccaagcctctcatcactaccctcctccctaagatgattgcccgcatcaacgactggtttgaggaagtgaaagctaagcgcggcaggcgcccgacagccttcaagttcctcaaagaaatcaagccggaagccgtagcgtacatcaccattaagacctctctggcttgcctcaccagtgctgacaatacaaccgttcaggctgtagcaagcgcaatcggtcggaccattgaggacgaggctcgcttcggtcgtatccgtgaccttgaagctaagcacttcaagaaaaacgttgaggaacaactcaacaagcgcgtagggcacgtctacaagggtggatccggctctggttcgagttctgtcgctcctgagaccgtgggctctacaagtgtggtcgcagtggtcttcccatctcacatttttgtcgcaaattgcggcgacagccgggccgtgctgtgcaggggaaagaccgccctgccactgagcgtggaccataaacccgacagggaggatgaagcagcccgcatcgaggctgcaggaggcaaagtgatccagtggaacggagcacgggtgtttggcgtcctggccatgtcacgcagcattggggatcgatacctgaaaccatcaatcattcccgaccctgaagtgactgccgtcaagagagtgaaagaagacgattgcctgatcctggctagcgacggcgtctgggatgtgatgaccgacgaggaagcatgtgagatggcccgaaagcggattctgctgtggcataagaaaaatgccgtggctggcgatgctagtctgctggcagacgagcggagaaaggaagggaaagatcccgccgctatgagtgcagccgaatatctgtcaaaactggctattcagaggggcagtaaggacaacatctccgtcgtcgtcgtggacctgaagggcggtagtggttcgggttcttcggtccccctgtatgggttcaccagcatttgtgggcggagaccagagatggaggcagccgtcagcactattccccggttcctgcagtcaagctccggcagcatgctggacgggaggttcgatccacagtccgccgctcacttctttggggtctacgatggacatggcgggtcccaggtggccaactattgcagggagcgcatgcacctggctctggcagaggaaatcgccaaggagaaacctatgctgtgcgacggagatacatggctggaaaagtggaagaaagccctgttcaactcttttctgcgggtggactccgagatcgaatgaggtaccattcaagacccccgcaccgaaaggtccgggggttttttttactatttaaatcctgccagtgttcacattcgaaccgtctctgctttgacaacatgctgtgcggtgttgtaaagtcgtggccaggagaatacgacaggcaattgcaagaaggaggatattgatgccatcggaacttacaccggaggaaaggtccgagttgaagaacagcattgcggagtttcatacatatcaactcgatccggggagttgttctagtttgcacgcccaaaggatacacgccccaccggaacttgtgtggagtatcgtccgccggttcgataaaccacaaacacacaggcatttcattaaaagctgctccgtggagcagaactttgaaatgagagtgggatgcacacgggatataatagttataagcggcctcccggccaacacgagcaccgaaagattggacatactggatgacgagcgccgcgtgacgggggcatcaataatcggtggggaacatcgccttacgaattataaaggcgtcactactgttcatagatttgagaaggaaaatagaatctggactgttgttctggaatcttatgtggttgatatgccagagggtaactcggaagatgatacacgcatgctggcggacacggttgtgaaacttaatctgcaaaagctggcaactgtcgccgaagccatggcaggtagcgcaggatcaggctcgagtggtaaagcatttatgcaagttgtcgaggctgacatgctctctaagggtctcctcggtggcgaggcgtggtcttcgtggcataaggaagactctattcatgtaggagtacgctgcatcgagatgctcattgagtcaaccggaatggttagcctccaccgccaaaatgctggcgtagtaggtcaagactctgagactatcgaactcgcacctgaatacgctgaggctatcgcaacccgtgcaggtgcgctggctggcatctctccgatgttccaaccttgcgtagttcctcctaagccgtggactggcattactggtggtggctattgggctaacggtcgtcgtcctctggcgctggtgcgtactcacagtaagaaagcactgatgcgctacgaagacgtttacatgcctgaggtgtacaaagcgattaacattgcgcaaaacaccgcatggaaaatcaacaagaaagtcctcgcggtcgccaacgtaatcaccaagtggaagcattgtccggtcgaggacatccctgcgattgagcgtgaagaactcccgatgaaaccggaagacatcgacatgaatcctgaggctctcaccgcgtggaaacgtgctgccgctgctgtgtaccgcaaggacaaggctcgcaagtctcgccgtatcagccttgagttcatgcttgagcaagccaataagtttgctaaccataaggccatctggttcccttacaacatggactggcgcggtcgtgtttacgctgtgtcaatgttcaacccgcaaggtaacgatatgaccaaaggactgcttacgctggcgaaaggtaaaccaatcggtaaggaaggttactactggctgaaaatccacggtgcaaactgtgcgggtgtcgataaggttccgttccctgagcgcatcaagttcattgaggaaaaccacgagaacatcatggcttgcgctaagtctccactggagaacacttggtgggctgagcaagattctccgttctgcttccttgcgttctgctttgagtacgctggggtacagcaccacggcctgagctataactgctcccttccgctggcgtttgacgggtcttgctctggcatccagcacttctccgcgatgctccgagatgaggtaggtggtcgcgcggttaacttgcttcctagtgaaaccgttcaggacatctacgggattgttgctaagaaagtcaacgagattctccaagcagacgcaatcaatgggaccgataacgaagtagttaccgtgaccgatgagaacactggtgaaatctctgagaaagtcaagctgggcactaaggcactggctggtcaatggctggcttacggtgttactcgcagtgtgactaagcgttcagtcatgacgctggcttacgggtccaaagagttcggcttccgtcaacaagtgctggaagataccattcagccagctattgattccggcaagggtctgatgttcactcagccgaatcaggctgctggatacatggctaagctgatttgggaatctgtgagcgtgacggtggtagctgcggttgaagcaatgaactggcttaagtctgctgctaagctgctggctgctgaggtcaaagataagaagactggagagattcttcgcaagcgttgcgctgtgcattgggtaactcctgatggtttccctgtgtggcaggaatacaagaagcctattcagacgcgcttgaacctgatgttcctcggtcagttccgcctccagcctaccattaacaccaacaaagatagcgagattgatgcacacaaacaggagtctggtatcgctcctaactttgtacacagccaagacggtagccaccttcgtaagactgtagtgtgggcacacgagaagtacggaatcgaatcttttgcactgattcacgactccttcggtaccattccggctgacgctgcgaacctgttcaaagcagtgcgcgaaactatggttgacacatatgagtcttgtgatgtactggctgatttctacgaccagttcgctgaccagttgcacgagtctcaattggacaaaatgccagcacttccggctaaaggtaacttgaacctccgtgacatcctcgagtcggacttcgcgttcgcgtaatagcaccaccaccaccaccactgagatccggctgctaacaaagcccgaaaggaagctgagttggctgctgccaccgctgagcaataactagcataaccccttggggcctctaaacgggtcttgaggggttttttgctgaaaggaggaactatatccg |
| pCDF-T7-mcherry  (Turquoise:  T7 promoter  yellow:  mcherry  purple: Terminator) | agatctcgatcccgcgaaattaatacgactcactatagggagaccacaacggtttccctctacaaataattttgtttaactttaagaaggagatatacatatggtctccaagggcgaggaggacaacatggccatcatcaaggagttcatgcgcttcaaggtccacatggagggctccgtcaacgggcacgagttcgagatcgagggcgagggggagggccggccgtacgagggcacccagaccgccaagctgaaggtgaccaagggcggccccctcccgttcgcctgggacatcctctccccccagttcatgtacggctcgaaggcatacgtcaagcacccggccgacatcccggactacctgaagctctcgttcccggaggggttcaagtgggagcgggtcatgaacttcgaggacggcggcgtcgtcaccgtcacccaggacagctccctgcaggacggcgagttcatctacaaggtcaagctgcggggcacgaacttcccgagcgacggccccgtgatgcagaagaagacgatgggctgggaagcgtcctcggagcgcatgtacccggaggacggcgccctcaagggcgagatcaagcagcgcctgaagctgaaggacggcggccactacgacgccgaagtcaagacgacgtacaaggccaagaagccggtgcagctcccggGAgcctacaacgtgaacatcaagctcgacatcacctcgcacaacgaggactacacgatcgtggagcagtacgagcgcgccgagggccggcactcgaccggcggcatggacgagctgtacaagtgagaattcttgttcagaacgctcggtcttgcacaccgggcgttttttctttgtgagtcca |
