# Supplementary material for: Agrochemical control of gene expression using evolved split RNA polymerase
Source: PeerJ. 2022 Jun 16;10:e13619. doi: 10.7717/peerj.13619 (PMC9206840; doi:10.7717/peerj.13619)
Supplement: Supplemental Information 13 [file peerj-10-13619-s013.docx]

|  | blank | 0 hour | 1 hour | 2 hour | 3 hour | 4 hour | 5 hour | 6 hour | 8 hour | 10 hour |
| --- | --- | --- | --- | --- | --- | --- | --- | --- | --- | --- |
| blank | 0.044 |  |  |  |  |  |  |  |  |  |
| blank | 0.042 |  |  |  |  |  |  |  |  |  |
| blank | 0.040 |  |  |  |  |  |  |  |  |  |
| Starting culture |  | 0.059 |  |  |  |  |  |  |  |  |
| Starting culture |  | 0.056 |  |  |  |  |  |  |  |  |
| Starting culture |  | 0.058 |  |  |  |  |  |  |  |  |
| control_1 |  |  | 0.070 | 0.123 | 0.330 | 0.493 | 0.709 | 0.906 | 1.008 | 0.059 |
| control_2 |  |  | 0.070 | 0.130 | 0.320 | 0.479 | 0.729 | 0.899 | 1.036 | 0.056 |
| control_3 |  |  | 0.071 | 0.129 | 0.324 | 0.480 | 0.711 | 0.891 | 1.000 | 0.058 |
| 100uM-1 |  |  | 0.070 | 0.121 | 0.316 | 0.475 | 0.711 | 0.893 | 0.988 | 0.994 |
| 100uM-2 |  |  | 0.070 | 0.128 | 0.325 | 0.481 | 0.716 | 0.937 | 1.039 | 0.993 |
| 100uM-3 |  |  | 0.070 | 0.129 | 0.320 | 0.484 | 0.709 | 0.888 | 0.997 | 0.990 |
| 400uM-1 |  |  | 0.080 | 0.121 | 0.292 | 0.414 | 0.626 | 0.857 | 0.959 | 0.955 |
| 400uM-2 |  |  | 0.068 | 0.133 | 0.323 | 0.476 | 0.679 | 0.893 | 0.997 | 0.969 |
| 400uM-3 |  |  | 0.069 | 0.141 | 0.333 | 0.485 | 0.691 | 0.913 | 1.008 | 1.005 |

TableS3: OD_600_ values of *E.coli* culture, related to supplemental figure 4.
