# Supplementary material for: Agrochemical control of gene expression using evolved split RNA polymerase
Source: PeerJ. 2022 Jun 16;10:e13619. doi: 10.7717/peerj.13619 (PMC9206840; doi:10.7717/peerj.13619)
Supplement: Supplemental Information 14 [file peerj-10-13619-s014.docx]

Table S4, percentage of GFP positive cells under unpaired two-tailed T-test.

|  | Position 1 | Position 2 | Position 3 |
| --- | --- | --- | --- |
| DMSO | 2.472 | 2.392 | 5.128 |
| Mandi | 55.480 | 66.389 | 64.634 |

mean fluorescence intensity of GFP positive cells under unpaired two-tailed T-test.

|  | Position 1 | Position 2 | Position 3 |
| --- | --- | --- | --- |
| DMSO | 21.272 | 18.919 | 17.135 |
| Mandi | 22.863 | 25.756 | 29.057 |
